# Body Size, Physical Activity and Risk of Colorectal Cancer with or without the CpG Island Methylator Phenotype (CIMP)

**DOI:** 10.1371/journal.pone.0018571

**Published:** 2011-04-05

**Authors:** Laura A. E. Hughes, Colinda C. J. M. Simons, Piet A. van den Brandt, R. Alexandra Goldbohm, Anton F. de Goeij, Adriaan P. de Bruïne, Manon van Engeland, Matty P. Weijenberg

**Affiliations:** 1 Department of Epidemiology, GROW School for Oncology and Developmental Biology, Maastricht University, Maastricht, The Netherlands; 2 Department of Prevention and Health, TNO Quality of Life, Leiden, The Netherlands; 3 Department of Pathology, GROW School for Oncology and Developmental Biology, Maastricht University Medical Centre, Maastricht, The Netherlands; Health Canada, Canada

## Abstract

**Background:**

We investigated how body size and physical activity influence the risk of the CpG island methylator phenotype (CIMP) in colorectal cancer (CRC).

**Methods:**

In the Netherlands Cohort Study (n = 120,852), risk factors were self-reported at baseline in 1986. After 7.3 years of follow-up, 603 cases and 4,631 sub-cohort members were available. CIMP status according to the Weisenberger markers was determined using methylation specific PCR on DNA from paraffin embedded tumor tissue. Hazard rate ratios (HR) and 95% confidence intervals for CIMP (27.7%) and non-CIMP (72.3%) tumors were calculated according to BMI, BMI at age 20, BMI change, trouser/skirt size, height, and physical activity.

**Results:**

BMI modeled per 5 kg/m^2^ increase was associated with both CIMP and non-CIMP tumors, however, HRs were attenuated when additionally adjusted for trouser/skirt size. Trouser/skirt size, per 2 size increase, was associated with both tumor subtypes, even after adjustment for BMI (CIMP HR: 1.20, 95%CI: 1.01–1.43; non-CIMP HR: 1.14, 95%CI: 1.04–1.28). Height per 5 cm was associated with both tumor sub-types, but HRs were attenuated when adjusted for body weight. BMI at age 20 was positively associated with increased risk of CIMP tumors and the association was significantly less pronounced for non-CIMP tumors (*P*-heterogeneity = 0.01). Physical activity was inversely associated with both subtypes, but a dose-response association was observed only for non-CIMP tumors (*P*-trend = 0.02).

**Conclusions:**

Body size, especially central adiposity, may increase the risk of both CIMP and non-CIMP tumors. Body fat at young age may differentially influence risk. Physical activity appears to decrease the risk of CRC regardless of these molecular subtypes.

## Introduction

It is well accepted that indicators of energy balance influence the risk of colorectal cancer (CRC). A high body mass index (BMI), waist circumference, and adult attained height are clear risk factors for CRC, while physical activity has been shown to be protective [1]. Although CRC is one of the best described cancers in terms of genetic and epigenetic events involved [2]–[5], little is known about how measures of anthropometry and physical activity are associated with different molecular subsets of this disease. Elucidating potential differences in risk between molecular subtypes of CRC may lead to a better understanding of CRC, treatment, and prevention. This is especially important as the global prevalence of overweight and obesity continues to rise.

A distinct characteristic of epigenetic instability in CRC is the CpG island methylator phenotype (CIMP), characterized by numerous promoter CpG island hypermethylated tumor suppressor- and DNA repair genes [6]–[9]. This in turn is associated with transcriptional silencing of gene expression [10]. Few studies have investigated associations between indicators of energy balance and CIMP status, and those that have, only considered BMI as an indicator of body size. In a case-control setting, Slattery *et al.* reported an association between a high BMI and CIMP low but not CIMP high colon tumors [11], and no association between BMI and CIMP status in rectal tumors [12]. Vigorous physical activity was associated with both CIMP high and CIMP low colon tumors, but not rectal tumors [11], [12]. It has been hypothesized that DNA methylation is a consequence of inflammation [13], [14]. Central adiposity is also associated with chronic inflammation [15]. Therefore, considering waist circumference as a risk factor for CIMP in addition to BMI is important. Additionally, methylation is thought to be an early event in CRC [16], so considering height and BMI at a young age may also be informative as these variables are indicative of *in utero* and early life exposures [17].

Using the prospective Netherlands Cohort Study on diet and cancer (NLCS), we investigated the association between BMI, clothing size (as a proxy for waist circumference) and physical activity and risk of developing a tumor characterized by CIMP. Furthermore, in attempt to elucidate if timing of exposure is important for modulating this risk, we also investigated associations according to BMI at age 20, BMI change, and adult-attained height.

## Results

Baseline and molecular characteristics are described in **Table 1**. Proportionally, CIMP CRC cases were more likely to be female, have a tumor in the proximal colon, and be older than non-CIMP cases.

**Table 1:** Characteristics of NLCS study participants according to colorectal cancer status after 7.3 years of follow-up.

	MEN			WOMEN		
	Sub-cohort *	CIMP+	Non-CIMP	Sub-cohort	CIMP+	Non-CIMP
***Baseline characteristics***						
Total (% of cases)	2219	83 (25)	245 (75)	2390	81 (30)	190 (70)
Age (years) †	61.3 (4.2)	63.0 (4.1)	62.8 (4.1)	61.5 (4.3)	63.3 (4.0)	62.8 (4.0)
Height (cm)	176.4 (6.7)	176.5 (6.7)	176.9 (6.6)	165.1 (6.2)	166.5 (6.2)	166.2 (6.3)
BMI (kg/m^2^)	25.0 (2.6)	25.8 (2.6)	25.3 (2.9)	25.1 (3.6)	25.4 (2.9)	25.6 (3.5)
BMI (kg/m^2^) at age 20	21.8 (2.4)	22.4 (2.5)	21.8 (2.2)	21.4 (2.8)	21.5 (2.2)	21.6 (2.6)
BMI change						
Clothing size	51 (4)	52 (6)	52 (3)	44 (3)	44(3)	44(3)
Recreational physical activity (%)						
<30 min/day	19	21	13	27	33	31
30–60 min/day	31	30	32	31	23	32
60–90 min/day	18	20	18	22	18	22
>90 min/day	31	29	37	21	28	15
Occupational physical activity (%)						
<8 kj/min	57	72	63	58	52	51
8–12 kj/min	27	18	22	36	40	40
>12 kj/min	16	11	15	6	8	10
Sports participation during youth (%)						
Never	50	52	49	62	73	62
Only after puberty	23	24	20	20	15	22
Before, during, and after puberty	27	24	31	19	12	16
***Molecular characteristics***						
Site (%)						
Proximal colon	-	54	20	-	67	25
Distal colon	-	24	39	-	18	37
Rectosigmoid	-	5	11	-	4	17
Rectum	-	17	30	-	11	22
MSI status (%)						
Stable	-	72	95	-	60	97
Unstable	-	28	5	-	40	3
BRAF V600E mutation status						
Wildtype	-	64	94	-	54	93
Mutated	-	36	6	-	46	7

*from subcohort of 5000 individuals selected randomly at baseline.

† mean (sd) or percentages where indicated.

Associations between measures of anthropometry, physical activity and CRC risk according to CIMP status are shown in **Table 2**. After adjustment for age and sex, BMI modeled per 5 kg/m^2^ increase was statistically significantly associated with CIMP tumors (HR: 1.29, 95% CI: 1.01–1.66), and a statistically significant dose-response relationship was observed when modeling quartiles of BMI (*P*-trend = 0.02). However, when models were mutually adjusted for trouser/skirt size, these associations were greatly attenuated. BMI was also positively, though not statistically significantly, associated with non-CIMP tumors. These were also attenuated when mutually adjusted for trouser/skirt size. The HRs for CIMP and non-CIMP tumors did not differ significantly from one another (*P*-heterogeneity = 0.78). BMI at age 20 modeled per 5 kg/m^2^ increase was associated with increased risk of both subtypes, but HRs did not reach statistical significance. When BMI at age 20 was modeled in quartiles, a statistically significant trend was observed for CIMP tumors (*P* = 0.03). This trend became borderline significant when the model was adjusted for trouser/skirt size (*P*-trend  = 0.07). The test for heterogeneity between tumor subtypes was statistically significant with respect to BMI at age 20, even after adjustment for trouser/skirt size (*P*-heterogeneity = 0.01). No statistically significant observations were observed with respect to BMI change.

**Table 2:** Hazard Ratios (HR) and 95% Confidence Intervals (CI) for colorectal cancer characterized by CIMP, according to measures of anthropometry in the NLCS after 7.3 years of follow-up.

			CIMP +			Non-CIMP				
	PY	N	HR (95%CI) *	HR (95%CI) †	HR (95%CI) ‡	N	HR (95%CI) *	HR (95%CI) †	HR (95%CI) ‡	*P*-heterogeneity §
**BMI at baseline**										
Per 5 kg/m^2^	24,272	117	1.29 (1.01–1.66)	1.25 (0.96–1.64)	1.04 (0.73–1.49)	318	1.19 (0.98–1.14)	1.19 (0.98–1.14)	1.08 (0.85–1.38)	
Quartiles										
1	8080	29	1 (referent)	1 (referent)	1 (referent)	92	1 (referent)	1 (referent)	1 (referent)	
2	4491	20	1.28 (0.71–2.30)	1.22 (0.67–2.23)	1.11 (0.62–2.00)	57	1.01 (0.71–1.45)	1.00 (0.69–1.43)	0.97 (0.67–1.40)	
3	7901	44	1.52 (0.94–2.44)	1.45 (0.90–2.35)	1.22 (0.73–2.04)	109	1.19 (0.89–1.59)	1.18 (0.88–1.59)	1.09 (0.79–1.50)	
4	3799	24	1.85 (1.07–3.21)	1.77 (1.01–3.11)	1.45 (0.90–2.35)	60	1.22 (0.86–1.75)	1.22 (0.84–1.75)	1.03 (0.69–1.54)	
*P*-trend			0.02	0.03	0.32		0.15	0.18	0.71	
										0.78
**BMI @ 20**										
Per 5 kg/m^2^	24,272	117	1.28 (0.96–1.72)	1.28 (0.96–1.72)	1.22 (0.90–1.65)	318	1.14 (0.92–1.39)	1.10 (0.90–1.34)	1.17 (0.94–1.44)	
Quartiles										
1	6112	21	1 (referent)	1 (referent)	1 (referent)	76	1 (referent)	1 (referent)	1 (referent)	
2	6139	24	1.17 (0.64–2.11)	1.23 (0.67–2.26)	1.16 (0.64–2.11)	91	1.20 (0.87–1.66)	1.21 (0.87–1.66)	1.24 (0.89–1.72)	
3	6070	42	2.01 (1.18–3.43)	2.09 (1.22–3.59)	1.94 (1.14–3.32)	74	0.99 (0.71–1.38)	0.98 (0.70–1.37)	1.02 (0.72–1.43)	
4	5951	30	1.53 (0.87–2.70)	1.57 (0.88–2.82)	1.43 (0.81–2.55)	77	1.08 (0.78–1.51)	1.04 (0.75–1.45)	1.12 (0.80–1.57)	
*P*-trend			0.03	0.03	0.07		0.95	0.81	0.8	
										0.01
**BMI change**										
<0	2896	10	0.61 (0.31–1.21)	0.63 (0.32–1.24)	0.52 (0.25–1.07)	28	0.72 (0.47–1.10)	0.74 (0.48–1.13)	0.65 (0.42–1.02)	
0–3.9	11,594	62	1 (referent)	1 (referent)	1 (referent)	158	1 (referent	1 (referent)	1 (referent)	
4–7.9	7709	34	0.77 (0.51–1.18)	0.74 (0.45–1.13)	0.89 (0.55–1.28)	102	0.94 (0.72–1.22)	0.94 (0.72–1.23)	1.00 (0.76–1.31)	
≥8.0	2073	11	0.93 (0.48–1.79)	0.86 (0.44–1.68)	1.06 (0.55–2.05)	30	1.11 (0.73–1.68)	1.08 (0.70–1.66)	1.23 (0.79–1.89)	
*P-*trend			0.89	0.86	0.37		0.28	0.37	0.09	
										0.93
**Trouser/skirt size at baseline**										
Per 2 sizes	24,272	117	1.20 (1.06–1.35)	1.20 (1.05–1.37)	1.20 (1.01–1.43)	318	1.14 (1.05–1.24)	1.14 (1.05–1.24)	1.15 (1.04–1.28)	
Quartiles										
1	9561	31	1 (referent)	1 (referent)	1 (referent)	104	1 (referent)	1 (referent)	1 (referent)	
2	7805	43	1.65 (1.03–2.64)	1.66 (1.05–2.69)	1.62 (0.99–2.65)	109	1.16 (0.84–1.61)	1.17 (0.88–1.56)	1.14 (0.85–1.54)	
3	4440	26	1.66 (0.98–2.83)	1.66 (0.97–2.85)	1.61 (0.87–2.97)	60	1.19 (0.86–1.65)	1.12 (0.80–1.58)	1.07 (0.74–1.54)	
4	2465	17	2.01 (1.10–3.67)	2.01 (1.07–3.77)	1.90 (0.86–4.17)	45	1.46 (1.04–2.04)	1.53 (1.04–2.24)	1.39 (0.87–2.23)	
*P*-trend			0.01	0.02	0.10		0.002	0.06	0.28	
										0.61
**Height at baseline**										
Per 5 cm	24,272	117	1.14 (1.00–1.30)	1.16 (1.01–1.33)	1.05 (0.91–1.22)	318	1.11 (1.01–1.21)	1.10 (1.00–1.21)	1.05 (0.95–1.17)	
Quartiles										
1	6589	28	1 (referent)	1 (referent)	1 (referent)	75	1 (referent)	1 (referent)	1 (referent)	
2	6294	31	1.17 (0.70–1.98)	1.19 (0.71–2.01)	1.06 (0.63–1.80)	81	1.16 (0.84–1.61)	1.16 (0.83–1.62)	1.10 (0.79–1.53)	
3	6612	32	1.18 (0.70–1.38)	1.19 (0.71–2.01)	0.99 (0.58–1.68)	85	1.19 (0.86–1.65)	1.13 (0.80–1.57)	1.08 (0.77–1.51)	
4	4777	26	1.38 (0.79–2.38)	1.47 (0.82–2.61)	1.05 (0.58–1.89)	77	1.46 (1.04–2.04)	1.45 (1.03–2.06)	1.24 (0.86–1.79)	
*P*-trend			0.28	0.23	0.96		0.04	0.06	0.31	
										0.98

*models adjusted for age and sex.

† models adjusted for age, sex, and additionally for family history of CRC (yes/no), smoking status (never smoker, ex-smoker, current smoker), socioeconomic status (level of education: primary school, junior high school, senior high school, higher vocational school, or university), total energy intake (kcal/day), alcohol intake (0, 0.1–4, 5–14, 15–29, ≥30 g/day), and consumption of red meat, fruit, vegetables, and grains (g/day). Models with anthropometric variables were additionally adjusted for baseline physical activity (based on recreational physical activity for women (low≤30 minutes/day, intermediate = 30–90 minutes/day, high≥90 minutes/day) and occupational physical activity at the longest held job for men (low≤8 kj/minute, intermediate  = 8–12 kj/minute, and high≥12 kj/minute).

‡ Models adjusted for age and sex, and mutually adjusted for other anthropometric variables. Models with BMI and BMI at age 20 were additionally adjusted for trouser/skirt size; the model for BMI change was additionally adjusted for BMI at age 20; the model with trouser/skirt size was additionally adjusted for BMI; the model with height was additionally adjusted for body weight.

§ P value for test that HR for two tumor subtypes are equal (based on model 3).

Trouser/skirt size, modeled per 2 unit size increase, was associated with both CIMP (HR: 1.20, 95%CI: 1.05–1.37) and non-CIMP tumors (HR: 1.14, 95% CI: 1.05–1.24) after multivariate adjustment, and these associations remained even when models were mutually adjusted for BMI. When trouser/skirt size was considered in approximate quartiles, the test for trend was significant for CIMP (p = 0.02) and borderline significant for non-CIMP (*P* = 0.06) tumors, although these were attenuated when models were mutually adjusted for BMI. Associations observed for CIMP and non-CIMP tumors were not statistically significantly different from each other (*P*-heterogeneity = 0.61).

Height, modeled per 5 cm increase was associated with both CIMP (HR: 1.16, 95% CI: 1.01–1.33) and non-CIMP (HR: 1.10, 95% CI: 1.00–1.21) tumors after multivariate adjustment. A borderline statistically significant trend was observed for risk of non-CIMP tumors when height was considered in quartiles (highest versus lowest quartile HR: 1.45, 95% CI: 1.03–2.06; *P* = 0.06) (p-heterogeneity = 0.98). These associations were attenuated when the model was mutually adjusted for body weight.

With low physical activity as the reference category, there was no inverse dose-response association between physical activity and CIMP tumors, although the HR for intermediate physical activity was statistically significant (HR: 0.50, 95%CI: 0.30–0.82) (**Table 3**). An inverse association was observed in a dose-response fashion for non-CIMP tumors (intermediate physical activity HR: 0.80, 95%CI: 0.61–1.04; high physical activity HR: 0.69, 95%CI: 0.47–0.96; *P*-trend = 0.02), however, associations with CIMP and non-CIMP tumors did not differ significantly from each other (*P*-heterogeneity = 0.33).

**Table 3:** Hazard Ratios (HR) and 95% Confidence Intervals (CI) for colorectal cancer characterized by CIMP, according to physical activity status in the NLCS after 7.3 years of follow-up.

	CIMP +				Non-CIMP			
	PY	N	HR (95%CI) *	HR (95%CI) †	N	HR (95%CI) *	HR (95%CI) †	*P*-heterogeneity §
**Physical activity** ||								
Low	9468	58	1 (referent)	1 (referent)	162	1 (referent)	1 (referent)	
Intermediate	9312	29	0.50 (0.30–0.82)	0.50 (0.30–0.81)	115	0.80 (0.61–1.04)	0.81 (0.61–1.07)	
High	4252	21	0.82 (0.49–1.38)	0.81 (0.48–1.36)	44	0.67 (0.47–0.96)	0.69 (0.47–1.01)	
*P*-trend			0.22	0.22		0.02	0.04	
								0.33

*models adjusted for age and sex.

† models adjusted for age, sex, and additionally for trouser/skirt size, family history of CRC (yes/no), smoking status (never smoker, ex-smoker, current smoker), socioeconomic status (level of education: primary school, junior high school, senior high school, higher vocational school, or university), total energy intake (kcal/day), alcohol intake (0, 0.1–4, 5–14, 15–29, ≥30 g/day), and consumption of red meat, fruit, vegetables, and grains (g/day).

§ P value for test that HR for two tumor subtypes are equal.

|| Physical activity variable is based on baseline non-occupational physical activity for females, and occupational physical activity in males, as described in the methods.

Associations for a tumor methylation index in relation to anthropometric risk factors and physical activity are shown in **Table 4**. There was no clear pattern with respect to the degree of methylation, however, when modeled per 2 unit size increase, trouser/skirt size was associated in a dose-response manner with tumors displaying the highest level of methylation (4–7 genes methylated HR: 1.29, 95%CI: 1.06–1.58; *P*-trend = 0.08).

**Table 4:** Hazard ratios (HR) * and 95% confidence intervals (CI) for a methylation index † according to anthropometric risk factors and physical activity in the NLCS 7.3 years of follow-up.

			0–1 genes methylated		2–3 genes methylated		4–7 genes methylated
	PY	N	HR (95% CI)	N	HR (95% CI)	N	HR (95% CI)
**BMI at baseline**							
Per 5 kg/m^2^	24,272	116	1.07 (0.74–1.54)	154	1.05 (0.75–1.48)	100	1.02 (0.67–1.56)
Quartiles							
1	8080	44	1 (referent)	42	1 (referent)	24	1 (referent)
2	4491	12	0.48 (0.24–0.97)	31	1.15 (0.70–1.90)	20	1.33 (0.71–2.50)
3	7901	40	0.85 (0.52–1.39)	50	1.20 (0.75–1.91)	37	1.15 (0.63–2.10)
4	3799	20	0.71 (0.38–1.33)	31	1.37 (0.79–2.39)	19	1.19 (0.52–2.70)
P-trend			0.43		0.28		0.75
**BMI @ 20**							
Per 5 kg/m^2^	24,272	116	1.28 (0.95–1.73)	154	1.23 (0.93–1.63)	100	1.06 (0.75–1.49)
Quartiles							
1	6112	23	1 (referent)	37	1 (referent)	20	1 (referent)
2	6139	40	1.81 (1.07–3.06)	34	0.91 (0.56–1.46)	20	1.02 (0.54–1.92)
3	6070	24	1.07 (0.59–1.91)	39	1.06 (0.67–1.67)	38	1.84 (1.06–3.19)
4	5951	29	1.31 (0.75–2.29)	44	1.24 (0.80–1.94)	22	1.09 (0.59–2.03)
P-trend			0.82		0.28		0.31
**BMI change**							
<0 kg/m^2^	6115	9	0.54 (0.25–1.15)	15	0.70 (0.39–1.28)	8	0.58 (0.26–1.28)
0–3.9	5973	59	1 (referent)	88	1 (referent)	52	1 (referent)
4–7.9	6100	39	1.05 (0.68–1.63)	39	0.70 (0.47–1.03)	28	0.81 (0.51–1.29)
≥8.0	6085	9	0.99 (0.47–2.08)	12	0.91 (0.49–1.72)	12	1.34 (0.69–2.57)
			0.28		0.63		0.35
**Trouser size at baseline**							
Per 2 sizes	24,272	116	1.13 (0.96–1.34)	154	1.12 (0.97–1.29)	100	1.29 (1.06–1.58)
Quartiles							
1	9561	48	1 (referent)	55	1 (referent)	28	1 (referent)
2	7805	26	0.68 (0.40–1.14)	56	1.25 (0.84–1.87)	33	1.52 (0.86–2.68)
3	4440	25	1.06 (0.62–1.81)	24	0.90 (0.52–1.56)	24	1.79 (0.88–3.64)
4	2465	17	1.24 (0.59–2.58)	19	1.22 (0.63–2.38)	15	2.00 (0.83–4.80)
P-trend			0.56		0.87		0.08
**Height at baseline**							
Per 5 cm	24,272	116	1.04 (0.89–1.22)	154	1.11 (0.96–1.28)	100	1.07 (0.92–1.24)
Quartiles							
1	6589	30	1 (referent)	36	1 (referent)	19	1 (referent)
2	6294	26	0.89 (0.52–1.51)	38	1.09 (0.68–1.74)	32	1.61 (0.91–2.88)
3	6612	33	1.06 (0.63–1.80)	41	1.11 (0.70–1.78)	27	1.23 (0.67–2.26)
4	4777	27	1.17 (0.65–2.11)	39	1.34 (0.80–2.25)	22	1.31 (0.67–2.55)
P-trend			0.52		0.30		0.68
**Physical activity** ‡							
Low	9468	56	1 (referent)	81	1 (referent)	47	1 (referent)
Intermediate	9312	48	0.85 (0.55–1.31)	56	0.82 (0.57–1.18)	28	0.60 (0.37–1.02)
High	4252	18	0.72 (0.41–1.28)	17	0.54 (0.31–0.94)	18	0.88 (0.50–1.54)
p-trend			0.25		0.02		0.42

*All models were adjusted for age and sex. Models with BMI and BMI at age 20 were additionally adjusted for trouser/skirt size, models with BMI change were additionally adjusted for BMI at ahe 20, models with trouser/skirt size were additionally adjusted for BMI, and models with height were additionally adjusted for body weight.

† methylation index including the five CIMP markers (*CACNA1G, IGF2, NEUROG1, RUNX3, and SOCS1*), *MLH1*, and the *APC* gene.

‡ Based on baseline non-occupational physical activity for women (low≤30 minutes/day, intermediate = 30–90 minutes/day, high≥90 minutes/day) and occupational physical activity at the longest held job for men (low≤8 kj/minute, intermediate  = 8–12 kj/minute, and high≥12 kj/minute).

## Discussion

These data suggest that adult body fatness and height may increase the risk of CRC, but are not differentially associated with CIMP and non-CIMP tumors. Contrarily, BMI at age 20 may be a stronger risk factor for CIMP+ tumors. Baseline physical activity appears to decrease the risk of CRC regardless of CIMP status.

A major strength of this study is that we investigated associations in a prospective cohort setting. The NLCS has almost complete ascertainment of colorectal cancer and very little loss to follow-up. Although the number of total CRC after 7.3 years of follow-up in the NLCS was substantial, the number of cases with the CIMP phenotype was small. With limited power to detect associations, it is possible that some findings arose by chance. Another potential limitation of this study is that anthropometric variables were obtained by self-report. However, there are many examples in the literature showing that this method is a valid and reliable tool for assessing body weight and height in cohort studies [18]–[21].

To our knowledge, associations between indicators of energy balance and CIMP status of colorectal tumors have been reported only by Slattery *et al.* in a case-control setting [11], [12]. In addition to the study design, there are differences between our studies which should be taken into account when comparing results.

The NLCS utilized the Weisenberger panel of genes to define CIMP (*CACNA1G, IGF2, NEUROG1, RUNX3* and *SOCS1*), whereas the study of Slattery *et al.*, utilized the Classic panel (*MINT1, MINT2, MINT31, p16 and hMLH1*), as well as different cut-offs to define CIMP in tumors [22], [23]. The ‘right’ definition of CIMP is still a highly debated topic, as is the ideal gene panel and the appropriate method of methylation detection [12], [24]. While the Weisenberger panel has been validated, different markers of methylation may be more or less informative with respect to studying different exposures. The prevalence of CIMP in the NLCS population is higher than in the study by Slattery *et al*. (27% vs. 11%) [12]. However, a difference in primer designs and PCR conditions may substantially change sensitivity and specificity of a particular marker for the detection of CIMP in CRC [25]. Therefore, it is likely that differences in prevalence are not due to the different methods per se, but rather a difference in choice of primers. The MSP analyses that have been conducted in the NLCS have a high detection signal, and subsequently, a higher prevalence of CIMP has been observed. In the present study, we attempted to clarify our observations by constructing a methylation index with different cut-off points that included seven genes commonly methylated in CRC.

In the present study we considered colon and rectal tumors together to increase statistical power. A sensitivity analysis revealed that this did not bias our findings (data not shown). Furthermore, we suggest that idea of combining sub-localizations of tumors may be acceptable when studying molecular endpoints, because this may help explain differences in etiology according to sub-localization.

We observed that BMI was associated with both tumor CIMP and non-CIMP tumors; however, after adjustment for clothing size, these associations disappeared. This is in contrast to case-cohort data suggesting that BMI is associated only with CIMP negative colon tumors and not with rectal tumors [11], [12]. In our study, trouser/skirt size appears to be a strong, independent predictor of both tumor subtypes, even after adjustment for BMI. This is logical, because waist circumference, an indicator of central adiposity, is a stronger predictor of CRC than BMI [1], [26]. When we considered associations according to a methylation index, we did not observe clear associations with respect to BMI and degree of promoter methylation, however, we did observed that trouser/skirt size was associated with the highest level of methylation. That we observed associations between trouser/skirt size and both CIMP and non-CIMP tumors suggests that central adiposity may influence CRC risk through both a methylation and a non-methylation pathway.

Although very few studies have considered associations between BMI and CIMP, a number have considered endpoints in the same pathway as CIMP. Colorectal cancer has distinct molecular subsets, which evolve through different pathways [16]. The path to a serrated adenocarcinoma appears to take one of two major routes: the traditional serrated pathway or the sessile serrated pathway [27]. The sessile serrated pathway is characterized by a high degree of CIMP, *BRAF* V600E mutations, and ultimately develops into microsatellite instability (MSI) [27]. MSI may serve as a marker for CIMP or other molecular events in CRC [28], therefore, it may be informative to consider the findings of the present study in the context of that research. Two case-control studies have reported that BMI appears associated with microsatellite stable (MSS) tumors, and less with MSI tumors [29], [30]. Neither study reported associations according to waist circumference. In a recent pooled analysis of NLCS and data from the Melbourne Collaborative Cohort Study, we observed similar associations, although the test for heterogeneity between the tumor subtypes was not statistically significant (Hughes *et al.*, submitted).

There is evidence to suggest that early life exposures influence epigenetic mechanisms associated with adult disease risk [31], [32]. Therefore, we also investigated associations between BMI at age 20, BMI change, height and CIMP status. Height is a marker of an aggregated fetal and childhood experience, and can be considered a proxy measure for important nutritional exposures, which affect several hormonal and metabolic axes [17]. In the NLCS, we have observed that childhood and adolescent energy restriction is associated with a decreased risk of CRC later in life [33], [34], which is supported by other population based studies [35]–[37]. We also recently reported that exposure to severe energy restriction during childhood and adolescence was associated with a low risk of developing a CIMP tumor [32]. Furthermore, pooled data suggest that taller individuals are at greater risk of developing a MSI tumor (Hughes *et al*. submitted). According to the present study, height is not differentially associated with the risk of tumors, however, we did observe significant heterogeneity with respect to BMI at age 20 and tumor subtypes. Taken together, our findings suggest that body size may differentially influence CIMP status during different periods of life, potentially affecting later CRC risk. The association between BMI at age 20 and CIMP tumors was stronger than with non-CIMP tumors, which is in line with previous findings for severe energy restriction during childhood and adolescence. Although our bootstrapping method is quite conservative, we did not observe a clear association with respect to BMI at age 20 and the methylation index and therefore we can not rule out that the differential association with CIMP status is a chance finding. The hypothesis that timing of exposure may influence epigenetic mechanisms requires further investigation.

That we did not observe any clear associations between BMI change and risk of tumors was surprising. This may indicate that metabolic changes in fat may be more important for modulating risk over time, rather than BMI. Alternatively, considering men and women together may have attenuated these observations. Campbell *et al.* report that adult weight gain was associated with CRC in men, but not in women, and only with respect to individuals who gained more than 21 kg since age 20 [29]. Finally, only considering two time points may not be indicative of true BMI change.

Our findings with respect to physical activity support those of Slattery *et al.* [11], and suggest that high levels of daily exercise are associated with a decreased risk of both CIMP and non-CIMP tumors. Observations with respect to our methylation index suggest that physical activity may be more protective of tumors with increasing methylation, but more research is required before firm conclusions can be drawn.

Preliminary evidence suggests that molecular markers can be used to classify colorectal cancers into distinct subtypes, which have implications for both etiology and prevention [28]. Fewer tumors arise from the sessile serrated pathway compared to the traditional adenoma pathway [27], [38]. As overweight and obesity are such strong risk factors for CRC, there is a possibility that these conditions may differentially influence risk through pathways and molecular mechanisms other than what we investigated here. More research is needed to clarify the association between indicators of energy balance and epigenetic mechanisms leading to CRC; preferably in a prospective cohort setting, with many cases [39]. Furthermore, as the field of molecular pathological epidemiology [40] continues to evolve, standardizing methods and definitions of molecular endpoints should be addressed. This will become especially critical as more opportunities for pooling data arise.

In conclusion, our findings suggest that measures of anthropometry reflecting a large body size increase the risk of both CIMP and non-CIMP tumors, and that body fat at young age may differentially influence risk. Physical activity appears to decrease the risk of CRC regardless of these molecular subtypes. Our observations reiterate the importance of a healthy body weight with respect to general CRC prevention.

## Materials and Methods

### Study populations and design

The NLCS is a prospective cohort study that was initiated in 1986 to investigate the association between diet and the development of cancer. It includes 58,279 men and 62,573 women between the ages of 55–69 years at baseline who completed a self-administered food frequency questionnaire involving 150 food items as well as questions on dietary habits, lifestyle, health, and demographics. Municipal registries from throughout the Netherlands were used to constitute an efficient sampling frame. The NLCS uses a case – cohort approach for data processing and analysis; case subjects were derived from the entire cohort, and the number of person-years at risk for the entire cohort was estimated from a sub cohort of 5000 men and women who were randomly sampled from the full cohort at baseline. All sub cohort members who reported prevalent cancer (excluding skin cancer) at baseline were excluded from analyses, leaving 4654. Further details of the NLCS design have been described [41]–[43].

Incident CRC cases were identified by annual record linkage to nine regional cancer registries and a national pathology database (PALGA) [44]. The completeness of cancer follow-up is almost 100% [45]. Paraffin embedded tumor material from CRC patients was retrieved, as described previously [46]. In total, 734 incident CRC patients were identified from a follow-up period of 7.3 years after baseline, excluding the first 2 years of follow-up, of whom a PALGA report of the lesion as well as sufficient DNA was available [46].

The study protocol was approved by the Medical Ethics Committees of the University Hospital Maastricht and TNO Nutrition. On recruitment, participants were informed in writing of the details of the study and its objectives. In accordance with the regulations at that time, written informed consent was obtained when participants returned the completed baseline questionnaire. Tumor material was collected after approval by the ethical review boards of Maastricht University, the National Cancer Registry, and PALGA.

### Ascertainment of risk factors

#### Anthropometric variables

Height (cm), body weight (kg), and body weight at age 20 (kg) were self-reported on the baseline questionnaire. From these variables, BMI and BMI at age 20, and BMI change were subsequently calculated. At baseline, individuals were also asked to report their lower body (trouser or skirt) clothing size from their clothing label (Dutch sizes). Trouser/skirt size has been shown to be an adequate proxy measure for waist circumference when predicting cancer risk in the NLCS, and details of how clothing size corresponds to waist measurements in men and women in this Dutch population has been published [47]. BMI, BMI at age 20, skirt/trouser size, and height were categorized into approximate sex-specific quartiles. As in previous NLCS analyses, BMI change was categorized as: <0 kg/m^2^, 0–4 kg/m^2^, 4–8 kg/m^2^, and >8 kg/m^2^ [48].

#### Physical activity

With respect to physical activity and CRC risk in the NLCS population, occupational physical activity appears to be more important for men and recreational physical activity for women for predicting risk (Simons *et al.*, submitted). Therefore, we used these two variables to create sex-specific categories of ‘low,’ ‘intermediate’ and ‘high’ physical activity.

Occupational physical activity was derived from data on participants' occupational history. Using information on the type of job and the duration, energy expenditure and sitting time was calculated for the longest and last held job. Energy expenditure was based on a rating system developed by Hettinger *et al.* [49] and distinguishes between work of low, moderate and high activity which corresponds to an energy expenditure of <8, 8–12 and >12 kJ/min. Men were categorized into the ‘low’ category if their occupational physical activity was <8 kj/minute, ‘intermediate’ if they fell into the 8–12 kJ/minute category, and high if their occupational physical activity was >12 kJ/minute.

Baseline non-occupational physical activity was calculated based on two questions. The first (open-ended) question was ‘How many minutes do you spend on average per day walking or cycling to your work, to go shopping or to take out your dog?’ The reported time spent per day was categorized into ≤10, >10–30, >30–60 and >60 minutes per day. The second question was ‘How many hours of your leisure time do you spend on average per week on 1) recreational cycling, walking, 2) gardening/doing odd jobs and 3) sports, gymnastics?’ Answering possibilities were never, <1 hour per week, 1–2 hours per week and >2 hours per week. The time spent on these activities and the minutes spent per day on walking or cycling to work, to go shopping or to take out the dog were summed to obtain an overall measure of baseline non-occupational physical activity, with categories <30, >30–60, >60–90 and >90 minutes per day. Low physical activity was defined as <30 minutes/day, intermediate as 30–90 minutes/day, and high as >90 minutes/day.

### Promoter Methylation Analyses

CIMP in tumor tissue of CRC cases was defined by CpG island promoter hypermethylation of at least 3 out of 5 methylation markers (*CACNA1G, IGF2, NEUROG1, RUNX3* and *SOCS1*), as proposed by Weisenberger *et al.* [9] were determined by bisulfite modification of 500 ng genomic DNA using a commercially available kit (Zymo Research), and subsequent methylation specific PCR (MSP) [50], [51]. We chose to use MSP as a method because it is effective, specific and does not require specific equipment. It has been shown that results from MSP are in accordance with other technologies, such as MethyLight [52]. Additionally, the methylation status of two other genes, *APC* and *MLH1*, were determined and we added them to the CIMP markers to create a methylation index of seven genes.

To facilitate MSP analysis on DNA retrieved from formalin-fixed, paraffin-embedded tissue, DNA was first amplified with flanking PCR primers that amplify bisulfite-modified DNA but do not preferentially amplify methylated or unmethylated DNA. The resulting fragment was used as a template for the MSP reaction. All PCRs were carried out with controls for unmethylated alleles (DNA from normal lymphocytes), methylated alleles [normal lymphocyte DNA treated *in vitro* with *Sss*I methyltransferase (New England Biolabs, Ipswich, MA)] and a control without DNA. Ten microliters of each MSP reaction was directly loaded on to nondenaturing 6% polyacrylamide gels stained with ethidium bromide and visualised under UV illumination. The MSP analyses were successful for 81%, 79%, 79%, 90%, 83%, 93%, and 93% out of the 734 cases for *CACNA1G, IGF2, NEUROG1, RUNX3, SOCS1*, *MLH1*, and *APC* respectively.

### Statistical analyses

Data were analyzed with Stata (version 10, Statacorp, College Station, TX, USA). Cox proportional hazards analysis using the case-cohort approach was used to obtain hazard ratios (HR) and 95% confidence intervals (CI) for the association between measures of anthropometry and physical activity and CRC characterized by CIMP status. To improve statistical power, we considered men and women together. Tests for effect modification by sex were not statistically significant. The proportional hazards assumption was tested using the scaled Schoenfeld residuals and visual inspection of the hazard curves. To account for the additional variance introduced by sampling the subcohort from the entire cohort, standard errors were estimated using the robust option. Statistical significance was tested at the 0.05 level.

For all anthropometric variables in question, three models were considered. The first was adjusted only for age and sex. The second was additionally adjusted for variables identified as being associated with both CRC and energy balance from previous literature. These included family history of CRC (yes/no), smoking status (never smoker, ex-smoker, current smoker), socioeconomic status (level of education: primary school, junior high school, senior high school, higher vocational school, or university), total energy intake (kcal/day), alcohol intake (0, 0.1–4, 5–14, 15–29, ≥30 g/day), physical activity (low, medium, high as previously described), and consumption of red meat, fruit, vegetables, and grains (g/day). Finally, models were mutually adjusted for other anthropometric variables. Models including BMI and BMI at age 20 were mutually adjusted for skirt/trouser size, BMI change was adjusted for BMI at age 20, trouser/skirt size was adjusted for BMI, and height was adjusted for body weight.

We modeled physical activity adjusted for age and sex, and additionally adjusted for trouser/skirt size, family history of CRC (yes/no), smoking status (never smoker, ex-smoker, current smoker), socioeconomic status (level of education: primary school, junior high school, senior high school, higher vocational school, or university), total energy intake (kcal/day), alcohol intake (0, 0.1–4, 5–14, 15–29, ≥30 g/day), clothing size, and consumption of red meat, fruit, vegetables, and fiber (g/day).

To assess how measures of anthropometry and physical activity were associated with the extent of promoter methylation in the CRC tumors, we used the aforementioned methylation index to categorize cases into one of three groups: ‘0–1 genes methylated’, ‘2–3 genes methylated’, or ‘4–7 genes methylated’. Of the 734 cases, 556 had sufficient information to be classified into one of the three categories. Models including anthropometric variables were adjusted for age, sex, and mutually adjusted for other anthropometric variables as previously described, and the model for physical activity was adjusted for age and sex.

Tests for heterogeneity were done to evaluate differences between subtypes of tumors (e.g., *CIMP vs. non–CIMP*) using the competing risks procedure in STATA. However, the SE for the difference of the log–hazard ratios from this procedure assumes independence of both estimated hazard ratios, which would overestimate that SE and thus overestimate the *P* values for their difference. Therefore, these *P* values and the associated confidence intervals were estimated based on a bootstrapping method that was developed for the case-cohort design, as described previously [53]. Each bootstrap analysis was based on 1000 replications.
